# Dataset for transient 3D simulations of turbulent premixed flames of Gas-to-Liquid (GTL) fuel

**DOI:** 10.1016/j.dib.2021.106956

**Published:** 2021-03-23

**Authors:** Abdellatif M. Sadeq, Samer F. Ahmed, Ahmad K. Sleiti

**Affiliations:** Department of Mechanical and Industrial Engineering, College of Engineering, Qatar University, P O Box 2713, Doha, Qatar

**Keywords:** CFD, Premixed turbulent combustion, Fan-stirred combustion bomb, Gas to liquids, GTL, Turbulent flame speed

## Abstract

A fan-stirred combustion vessel is used to study the premixed turbulent combustion of diesel, Gas to Liquids (GTL) and 50/50 diesel-GTL and to generate these datasets. A numerical simulation approach is implemented for modelling the premixed combustion of the three fuels under different thermodynamics and turbulence initial conditions, using Zimont Turbulent Flame Speed Closure (Zimont TFC) model. Different parameters are obtained from these simulation runs such as turbulent eddy viscosity (µ), turbulent kinetic energy (k), Damkohler number (Da), Reynolds number (Re_T_) and turbulent flame speed (S_t_). The raw, filtered and pre-processed data are imported from ANSYS Fluent and then listed on filtered tables for the ease of accessibility. These datasets can be then used to perform research in different related areas such as chemical kinetic mechanisms, ignition delay time, flame ignition mechanisms and flame extinction and diffusion. Also, they can be employed to further understand trends, patterns, and anomalies in data. In addition, they can be compared with other numerical models to establish a robust knowledge about the modelling of premixed turbulent combustion. For more information and discussion of the dataset creation, the reader is directed to the full-length article, “Abdellatif M. Sadeq, Samer F. Ahmed, Ahmad K. Sleiti, Transient 3D simulations of turbulent premixed flames of gas-to-liquid (GTL) fuel in a fan-stirred combustion vessel, Fuel, Volume 291, 2021, 120,184, ISSN 0016 2361, https://doi.org/10.1016/j.fuel.2021.120184” [1].

## Specifications Table

**Table:** 

Subject area	Fuel Technology
Specific subject area	Premixed turbulent combustion. GTL fuel
Type of data	Tables, images, Excel spread sheets
How data was acquired	Data were extracted from ANSYS Fluent 17.0 software and tabulated in a Microsoft Excel file
Data format	Raw, filtered, preprocessed
Parameters for data collection	Turbulent kinetic energy, turbulent flame speed, turbulent eddy viscosity, Reynolds number and Damkohler number
Description of data collection	These datasets are related to premixed turbulent combustion of diesel, GTL and 50/50 diesel-GTL blend using a cylindrical combustion bomb. Zimont model available in Fluent workbench was used for modelling the premixed turbulent combustion of the three fuels at different operating conditions, and to generate the raw data. These data were then extracted from Fluent and stored in a Microsoft Excel format, and they are available on the Mendeley data repository. The Microsoft Excel file contains four sheets; where the raw, filtered and preprocessed data related for the three fuels are tabulated. Parameters such as turbulent kinetic energy, turbulent eddy viscosity, turbulent flame speed, Reynolds number and Damkohler number were obtained at the corresponding equivalence ratio and turbulence intensities. After that, they were listed on filtering tables for the ease of accessibility.
Data source location	Institution: Qatar University City: Doha Country: Qatar
Data accessibility	Repository Name: Mendeley Direct URL to data: http://dx.doi.org/10.17632/ts2jd8zc9r.3
Related research article	Abdellatif M. Sadeq, Samer F. Ahmed, Ahmad K. Sleiti, Transient 3D simulations of turbulent premixed flames of gas-to-liquid (GTL) fuel in a fan-stirred combustion vessel, Fuel, Volume 291, 2021, 120,184, ISSN 0016 2361, https://doi.org/10.1016/j.fuel.2021.120184. [1]

## Value of the Data

• The data provides important turbulence parameters for diesel, GTL and diesel-GTL blends including turbulent eddy viscosity (µ), turbulent kinetic energy (k), Damkohler number (Da), Reynolds number (ReT) and turbulent flame speed (St). The data are useful to understand trends, patterns, and anomalies under a wide range of engine's operating conditions, allowing the use of each fuel under its optimum conditions and applications.
• The data can benefit researchers in different areas such as chemical kinetic mechanisms, ignition delay time, flame ignition mechanisms and flame extinction and diffusion. In addition, this data can contribute to remarkable improvements in different energy sector companies such as oil and gas production, pipeline and refining, mining companies, chemical refinements and renewable energy sectors.
• The data can be used by researchers and engineers to validate and compare different turbulence models and experimental data obtained using other techniques. In addition, the datasets can be used for conducting detailed analysis of turbulence statistics and energy spectrum fields.

## 1 Data Description

**Fig. 1:**
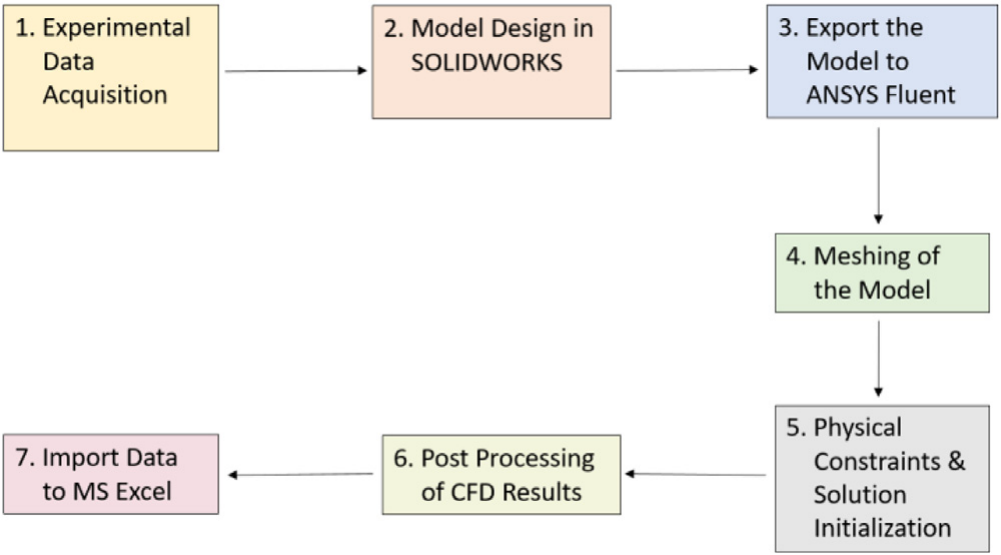
Steps followed for data curation.

This article presents datasets, which are related to premixed turbulent combustion of diesel, GTL and 50/50 diesel-GTL blend using a cylindrical fan-stirred combustion vessel. Zimont TFC model available in ANSYS Fluent 17.0 was used for modelling and simulation of the premixed turbulent flames of the three fuels at different operating conditions, and to generate the raw data. The flow chart in Fig. 1 shows the Steps used for data curation, starting from experimental data acquisition up to storing the data in MS Excel file.

**Fig. 2:**
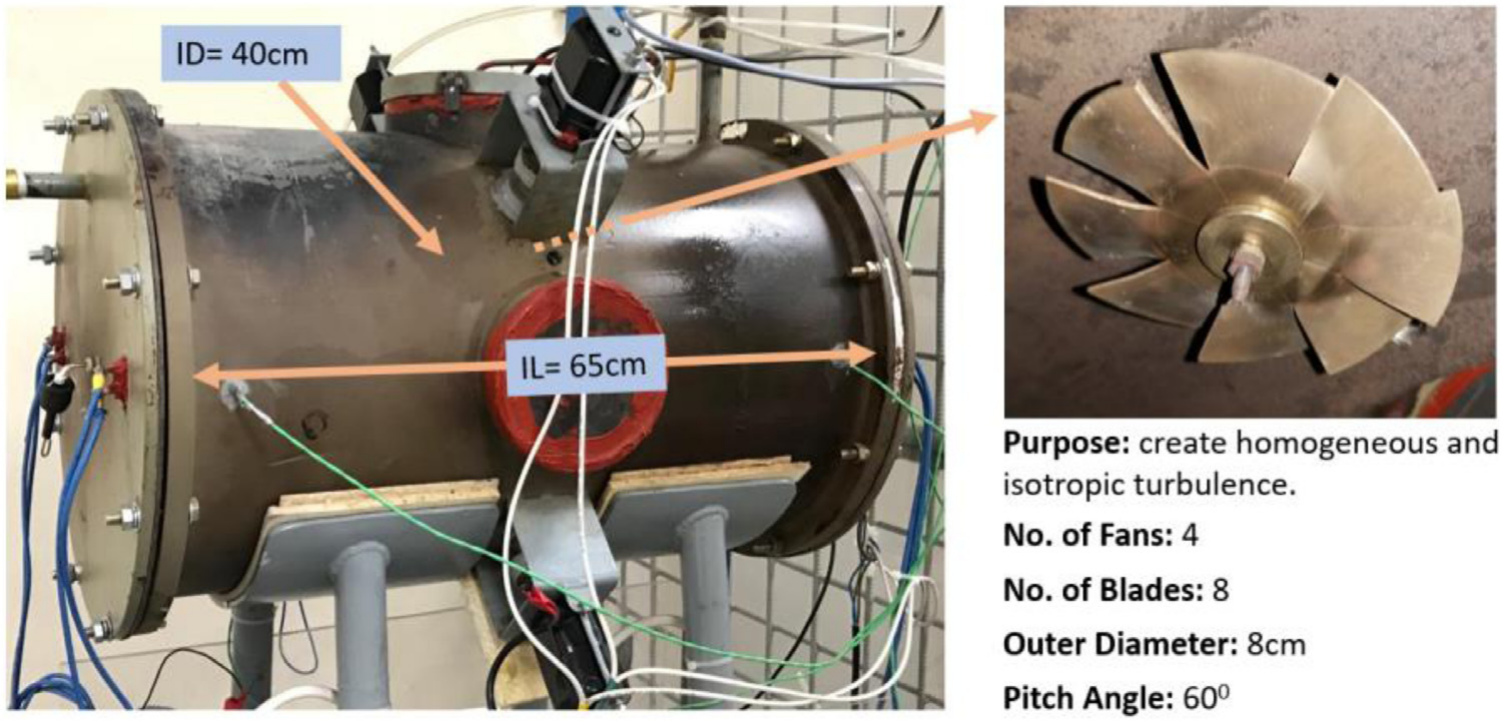
The main geometrical dimensions and specifications of QU vessel.

**Fig. 3:**
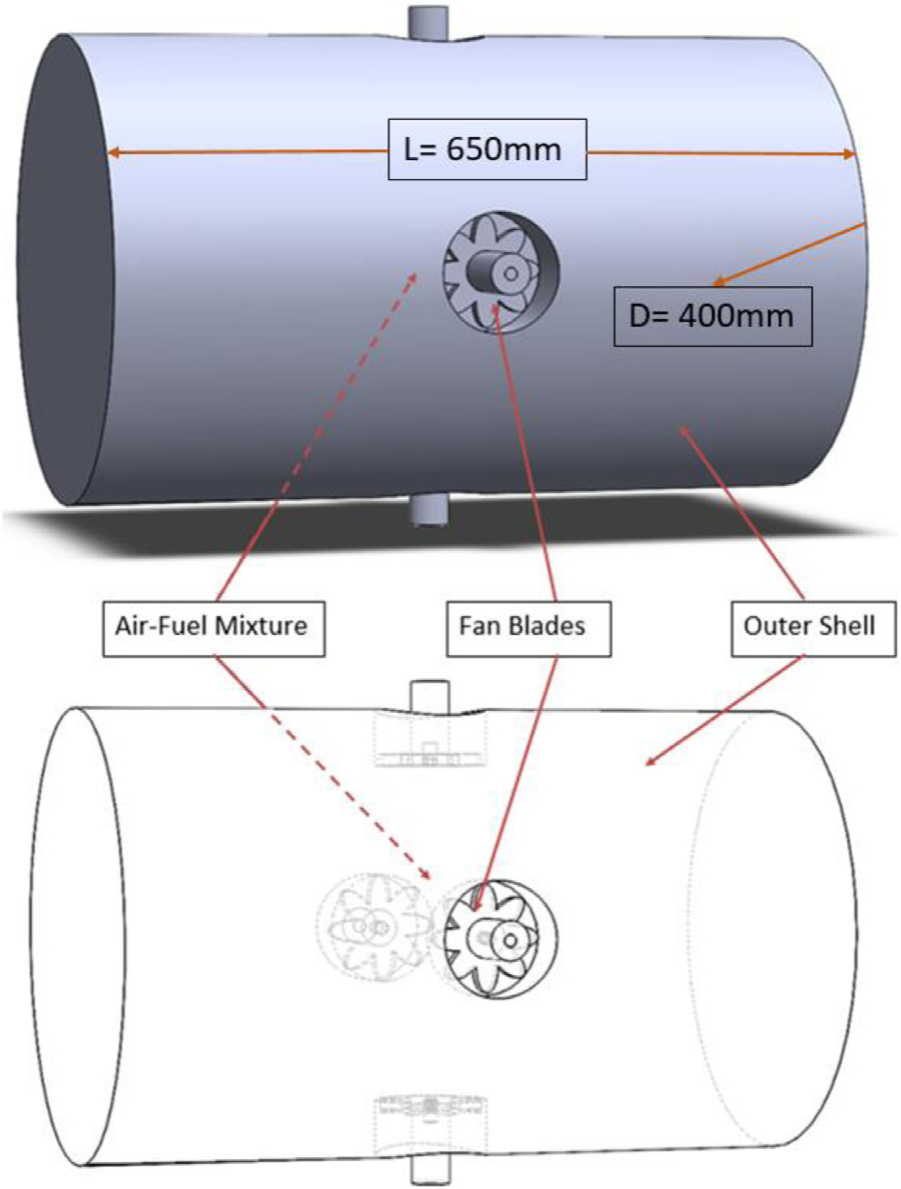
QU vessel's SOLIDWORKS model with basic geometrical dimensions and main components.

**Fig. 4:**
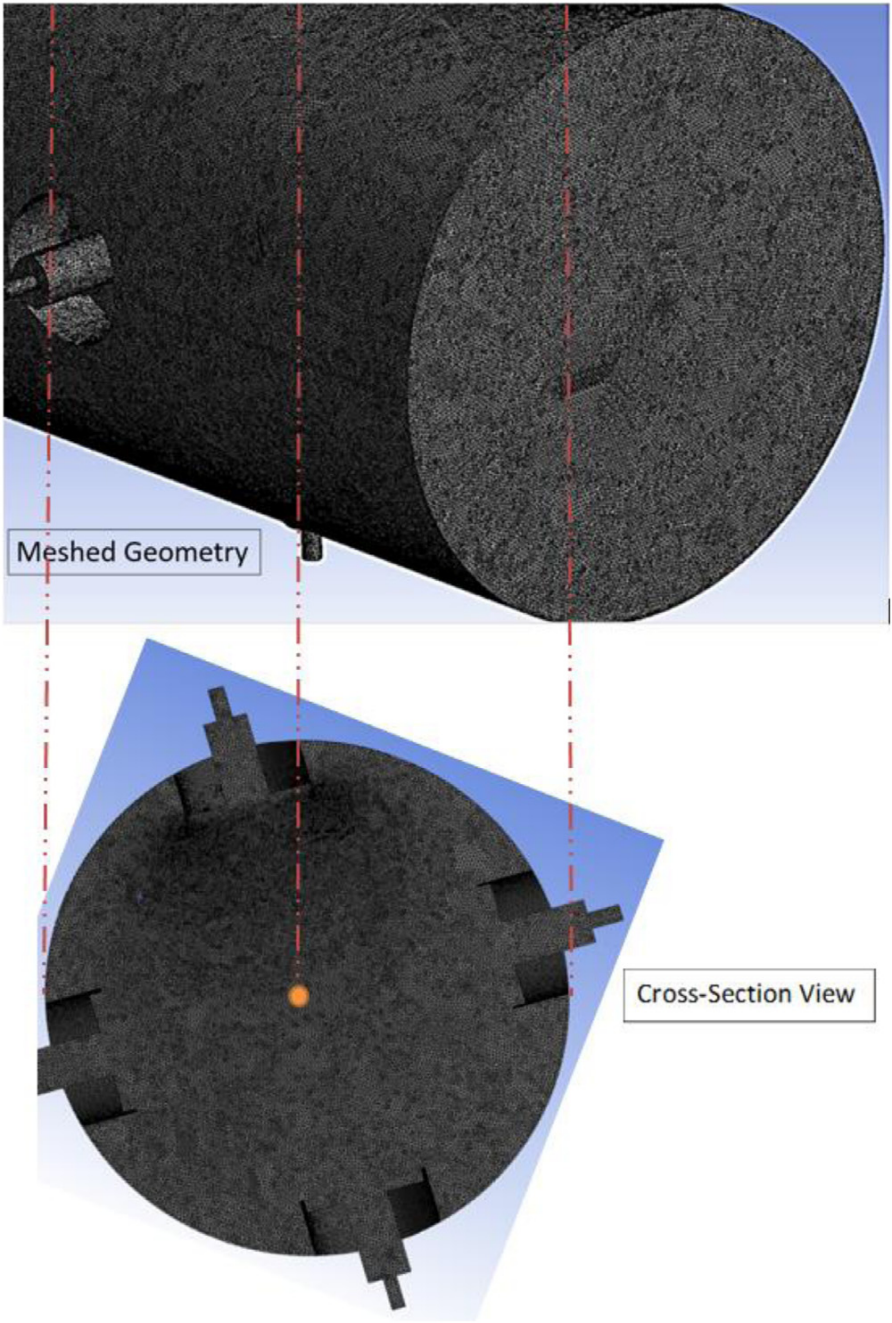
Perspective view of numerical grids on the combustion vessel surface.

The MS Excel file contains four sheets; where the raw, filtered and preprocessed data related for the three fuels are tabulated. Parameters such as turbulent kinetic energy, turbulent flame speed, turbulent eddy viscosity, Reynolds number and Damkohler number were obtained at the corresponding equivalence ratio and turbulence intensities, and then listed on filtering tables for the ease of use and access. In addition, this database allows obtaining the values of these parameters at any vessel radius up to 12 cm, and to find the flame radius at any time up to 30 ms. It should be noticed that all of these data were extracted when the elapsed time was equal to 30 ms, when the flame was slightly influenced by the turbulence domain and has left the ignition region. Raw data are available in the Mendeley Repository, and they are summarized in four tables. Table S1, S2 and S3 list the values of the premixed turbulent combustion parameters at the corresponding equivalence ratio and turbulence intensities for diesel, GTL and 50/50 diesel-GTL blend, respectively. Besides, Table S4 lists the values of flame radius as a function of time, equivalence ratio and turbulence intensity for the three fuels. In this article, Table 1 lists the thermodynamic and turbulence initial conditions used for modelling. Steps followed for data curation are listed in the flow chart of Fig. 1. The model's geometry, dimensions, and its main components are shown in Fig. 2. In Fig. 3, the basic geometrical dimensions and main components of QU vessel's SOLIDWORKS model are revealed, while Fig. 4 shows a perspective view of numerical grids on the combustion vessel surface.

**Table 1:** Thermodynamic and turbulence initial conditions used for modelling.

Fan Speed (RPM)	Turbulence Intensity,u` (m/s)	Turbulent Kinetic Energy, k (m^2^/s^2^)	Turbulence Dissipation Rate, ɛ (m^2^/s^3^)	Vessel Radius, r (cm)	Computed Parameters
**Φ=0.7 to 1.3, L_t_ =** 20 mm**, T_0_=** 463 K**, P_0_= 1atm**					

7000	0.50	0.38	2.31	0 to 12	µ, S_t_, *Re*, Da
9800	1.00	1.50	18.50	0 to 12	µ, S_t_, *Re*, Da
11,600	1.50	3.38	62.40	0 to 12	µ, S_t_, *Re*, Da
14,400	2.00	6.00	148.00	0 to 12	µ, S_t_, *Re*, Da
17,200	2.50	9.38	289.10	0 to 12	µ, S_t_, *Re*, Da
21,000	3.00	13.50	499.50	0 to 12	µ, S_t_, *Re*, Da

## 2 Experimental Design, Materials and Methods

A fan-stirred combustion vessel is used to study the premixed turbulent combustion of diesel, Gas to Liquids (GTL) and 50/50 diesel-GTL and to generate these datasets. This vessel has been designed at Qatar University, and its geometrical dimensions are used for modelling and obtaining the datasets. These type of vessels are the most commonly used geometry for turbulent flame speeds measurements [2]. Subsequently, this configuration is suitable for studying the premixed turbulent combustion of the three liquid fuels at various turbulent intensities and equivalence ratio. In this paper, the name “QU vessel “ indicates for using Qatar University combustion bomb for simplicity, and the main geometrical dimensions and specifications for the test rig are shown in Fig. 2. In addition, the reader is guided to Ref [3]. for more specifications and features about this test rig.

The physical, chemical and flame combustion parameters are estimated using CHEMKIN-PRO and GASEQ, which have extensive databases for kinetic, thermodynamics and transport parameters [4]. In addition, it should be noticed that mixture properties such as specific heat, viscosity, thermal conductivity are fixed to be constants in ANSYS Fluent. Although lean combustion (Φ<1.0) is more practically found in diesel engines operations, these datasets are generated at a wide flammability range (0.7 <Φ < 1.3) to clearly characterizing the behaviour of the flame under different operating conditions. The physical and chemical properties of the three fuels, in addition to flame combustion parameters are listed in Table 1 and Table 8 of Ref [1], respectively.

## 3 Numerical Simulation Approach

This section aims to present the numerical grid details, and to completely describe the method followed for meshing the SOLIDWORKS model, as specified in subsection 3.1. In addition, subsection 3.2 lists all the information related to the used numerical schemes, physical constraints, boundary conditions and solution initialization.

### 3.1 Numerical grid details

A cylindrical, steel vessel that has a diameter of 40 cm and a total volume of 81.7 L is considered for modelling in the problem geometrical domain. Four fans, which are used primarily to create a homogenous and isotropic turbulence are installed on the inner walls of the vessel. The axes of these fans are orientated collinearly with the vessel centre point, and the fans are placed on the vessel's central circumference while maintaining an equal distance from each other's (i.e., the axial distance between one fan to another equals to 30 cm). The outer diameter of each fan is 8 cm, and each fan has eight blades, each of which has a 3.5 cm length. For more specifications about the test rig, the reader is directed to Ref [3]. Also, it should be noticed that SOLIDWORKS 2020 has been used for designing the geometry, which has been later exported to ANSYS Workbench to complete the meshing and CFD post processing, and generate the datasets. Fig. 3 shows the basic dimensions of the combustion bomb model, and describes its main components.

The computational mesh domain consists of 7.8 million cells, and each cell has a 2 mm size. An adaptive size function is used to control the size of the elements, which are placed at an equal distance from each others while maintaining the tetrahedral shape. Fig. 4 shows a perspective view of numerical grids on the combustion vessel surface.

The resolution of the geometrical mesh domain has been specified through the relevance centre sizing option. It was set to be fine to achieve a more stable solution. In addition, the quality of numerical grids has been controlled and set to be “Medium” using the smoothing option, which can improve the numerical grids quality by placing the nodes at other locations with respect to other nodes or elements. Due to the high curvature found in the studied geometry, a fine span angle centre is suggested for use, aiming to span the curvature angle more precisely [5]. The numerical grid details used throughout the computational domain are listed in Table 3 of Ref [1].

### 3.2 Discretization and solution initialization

Turbulent flame speeds and flame radius evolution of the three investigated liquid fuels have been studied using Zimont TFC model. The range of equivalence ratio varies from *Ø*=0.7 to 1.3, and from u`= 0.5 m/s to 3.0 m/s for the turbulence intensity. The discretization scheme is the second order upwind, which can ensure a high accuracy of solution convergence while maintaining the insensitivity to grid size variation. The continuity and momentum equations are solved simultaneously using the coupled algorithm, which can result in a faster convergence to solution and a larger memory size consumption. In addition, it should be noticed that the mixture has to be specified as unburned prior to simulation start, thus a value of C=0 has to be specified in the solution initialization. The mixture is ignited in the centre of the vessel and the flame starts to propagate spherically in all the directions. Therefore, a spark plug with a 40mJ energy is placed in that location. The details of the used mixing charge, spark plug and numerical models are listed in Table 4 of Ref [1], whereas the information used in the solution initialization settings are listed in Table 5 of Ref [1].

The modelling has been conducted at an initial temperature of 463 K under atmospheric pressure. The variation of fan's speeds is used to obtain different turbulence intensities. For each equivalence ratio (Φ=0.7 to 1.3), fans speed have been varied from 7000RPM to 21000RPM (u`=0.50 m/s to u`=3.00 m/s), and parameters such as turbulent eddy viscosity, turbulent flame speed, Reynolds number and Damkohler number were computed. In addition, it should be noticed that these parameters have been computed for each of the three fuels, and listed for each one in a separate database. Table 1 lists all the thermodynamic and turbulence initial conditions at which the datasets were obtained.

## Ethics Statement

Ethical approval was obtained from the department of mechanical and industrial engineering at Qatar University, who have allowed accessing the simulation laboratory for using ANSYS Fluent 17.0 and SOLIDWORKS 2020 to generate the datasets.

## CRediT Author Statement

**Abdellatif Sadeq**: Methodology, Data curation, Resources, Validation, Investigation, Writing original draft; **Samer Ahmed:** Supervision, Project administration, Formal analysis, Writing review & editing; **Ahmad Sleiti:** Conceptualization, Methodology, Formal analysis, Supervision, Project administration, Writing review & editing.

## Declaration of Competing Interest

The authors confirm that they have no personal relationships or established conflicting financial interests that have influenced the research reported in this article, or might be perceived to have influenced it.
